# Comparison of Tissue Repair with Different Types of Microdissection Tips: A Randomized Histomorphometric Evaluation in Rats

**DOI:** 10.3390/bioengineering12070732

**Published:** 2025-07-04

**Authors:** Ana Luiza Vila Verde Brunelli, Luíz Henrique Soares Torres, Arthur Henrique Alécio Viotto, Izabela Fornazari Delamura, Ana Paula Farnezi Bassi, Marisa Aparecida Cabrini Gabrielli, Valfrido Antonio Pereira-Filho

**Affiliations:** 1 Department of Diagnosis and Surgery, School of Dentistry Araraquara, São Paulo State University (UNESP), Araraquara 14801-903, SP, Brazil; ana.brunelli@unesp.br (A.L.V.V.B.); marisa.gabrielli@unesp.br (M.A.C.G.); valfrido.pereira-filho@unesp.br (V.A.P.-F.); 2 Department of Diagnosis and Surgery, Araçatuba School of Dentistry, São Paulo State University (UNESP), Araçatuba 16015-050, SP, Brazil; luiz.torres@unesp.br (L.H.S.T.); izabela.delamura@unesp.br (I.F.D.); ana.bassi@unesp.br (A.P.F.B.)

**Keywords:** electrocoagulation, microdissection, surgical wound, healing, rats

## Abstract

The aim of the study was to compare tissue repair of incisions made using different microdissection electrocautery tips in an in vivo animal model. Skin incisions were made, including the subcutaneous tissue, in 30 adult Wistar rats using four types of instruments: a scalpel blade number 15, knife-type electrocautery, microdissection needle, and thin-cut electrode. The animals were divided into five groups based on the euthanasia time—24 h, 48 h, 72 h, 7 days, and 14 days. Each animal received four incisions, one with each type of instrument. Histological and histomorphometric analyses were performed using hematoxylin and eosin (HE) and Picrosirius red stains. Analysis of variance (ANOVA) showed that the type of dissector had no significant effect on type I collagen levels (*p* = 0.615), whereas the euthanasia time had a significant influence (*p* < 0.001). Estimated marginal means for type I collagen showed minimal variation among groups, ranging from 35.4% to 36.5%, suggesting limited clinical differences between instruments. These results indicate that while the choice of dissector has a limited impact on type I collagen deposition, time is a determining factor in the wound healing process. The thin-cut electrode enables incisions with tissue repair comparable to that of a number 15 scalpel, as it performs cutting, coagulation, and blending functions at lower temperatures.

## 1. Introduction

The process of tissue healing occurs in three interdependent and sequential phases: inflammation, proliferation, and remodeling (or maturation) [1,2]. Wound healing is a complex and dynamic process that involves the deposition and remodeling of collagen, an essential component of the extracellular matrix. Among the collagen types, type I and type III play fundamental roles in tissue repair. Type III collagen predominates in the early phases of healing, providing provisional support for cellular regeneration, while type I collagen is more abundant in later phases, conferring greater strength and stability to the scar matrix [3]. Therefore, the ratio of type I to type III collagen is an important indicator of the quality and maturation of healed tissue, directly reflecting the strength and functionality of the repaired wound. Alterations in these ratios have been associated with impaired healing and the formation of hypertrophic scars or keloids, highlighting their clinical relevance in wound prognosis and management [3,4]. The precision and care in performing tissue incisions are crucial for the functional and aesthetic outcome of healing [3].

A faster maturation of type I collagen is clinically desirable because it directly contributes to the mechanical strength and long-term stability of the healed tissue. Type I collagen, being the most abundant and structurally robust collagen type in mature scars, replaces the initially deposited type III collagen as healing progresses. This transition marks a critical phase in wound remodeling, as it enhances tensile strength and reduces the risk of wound dehiscence or pathological scarring [5]. Therefore, therapeutic strategies or surgical tools that promote early type I collagen deposition may accelerate tissue regeneration and improve the functional and aesthetic outcomes of wound healing [6].

Historically, conventional scalpel blades have been widely used in surgical procedures due to their precision, ease of handling, and relatively low cost [7]. However, these blades do not promote effective hemostasis, as they do not generate heat during the incision. In contrast, electrocautery, which uses electrical current for cutting and coagulation, provides efficient hemostasis, although it may induce thermal damage to the surrounding tissue if not properly controlled [8,9]. The primary goal of electrocautery is to enable precise incisions and efficient coagulation while minimizing lateral thermal dissipation [10].

The effectiveness of electrocautery is influenced by factors such as the intensity of the electrical current, contact time, and the diameter of the electrode tip. Electrodes with wider or blunt tips tend to generate greater thermal dispersion, which can lead to increased tissue necrosis [8,9,10]. In contrast, the microdissection needle, often used in oral and maxillofacial surgeries, features an extremely fine tungsten tip, allowing for more precise incisions with lower electrical power. This characteristic reduces the extent of tissue necrosis, enhances incision definition, and contributes to less postoperative pain [11,12].

Comparative studies [12,13] suggest that the microdissection needle may promote superior tissue repair when compared to conventional electrocautery tips, producing scar results similar to those observed with scalpel blades. However, financial limitations may restrict its adoption in certain countries.

More recently, even finer microdissection tips, known as “thin-cut” tips, have been developed with the aim of providing more delicate incisions and reducing lateral thermal dissipation [7,12,14]. However, the effectiveness of these innovations still requires detailed histological validation. Given the variety of microdissection tip options and their advantages, the present study aims to evaluate the tissue repair process after the use of different types of microdissection tips through histological and histomorphometric analyses.

## 2. Materials and Methods

This study was approved by the local Animal Use Ethics Committee (CEUA) (Process nº. 522/2023) and followed the protocol described for this type of experiment, according to ARRIVE (Animal Research: Reporting of In Vivo Experiments).

Thirty adult male rats, randomly separated (Rattus norvegicus albinus), Wistar variety, with an average body weight of 350 g and an age ranging from 3 to 4 months, were used. The animals were kept at the Bioterium of Arçatuba Dental School—UNESP in a controlled environment, in cages with shavings, normal feed (pellets or crushed), and water ad libitum. Surgical procedures followed the antisepsis and anesthesia protocols, using Ketamine Hydrochloride (Agener^®^ Ketamine, Agener União Ltd., São Paulo/SP, Brazil) 0.08 mL/100 g of body weight and 2% Xylazine (Rompun^®^ Bayer SA, São Paulo/SP, Brazil) 0.04 mL/100 g of body weight, both introduced by intraperitoneal injection. At the end of the procedure, the animals received antibiotics (Pentabiotic^®^, WyethWhitehall Ltd., São Paulo/SP, Brazil) and Ketoflex (1% ketoprofen, 0.03 mL/rat) intramuscularly.

**Figure 1:**
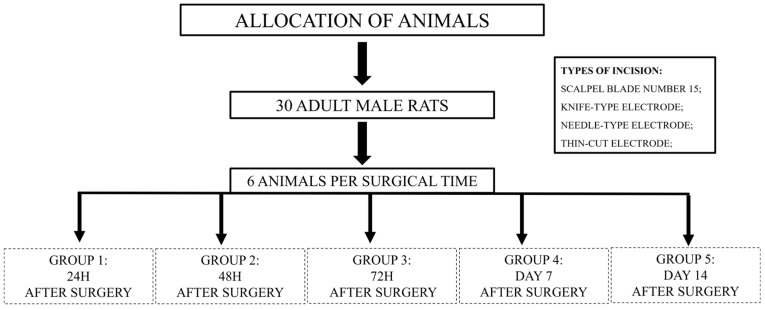
Animal allocation diagram by surgical time.

Six animals were separated for each surgical time, each of the samples received the four different types of incision: Group 1 (24 h after surgery); Group 2 (48 h after surgery); Group 3 (72 h after surgery); Group 4 (7 days after surgery); and Group 5 (14 days after surgery). The incisions were based on previously published work by this research group [13], using a scalpel blade number 15, a knife-type electrode, a needle-type electrode, and a thin-cut electrode. (Figure 1).

### 2.1. Surgical Procedure

Four 20-mm length incisions were made in each animal through the cutaneous and subcutaneous planes using the following instruments [15]:

Scalpel blade number 15 (Sterile Carbon Steel Scalpel Blade, Lamedid, Barueri/SP, Brazil): attached to the scalpel handle, the incisions were made according to length and depth, previously explained.

Knife-type electrode (BP-100 Plus, Emai Transmai, São Paulo/SP, Brazil) equipped with a conventional tip (67 mm).

Needle-type electrode (BP-100 Plus, Emai Transmai, São Paulo/SP, Brazil) equipped with a microdissection tip (52 mm) (Traumec, Rio Claro, Brazil).

**Figure 2:**
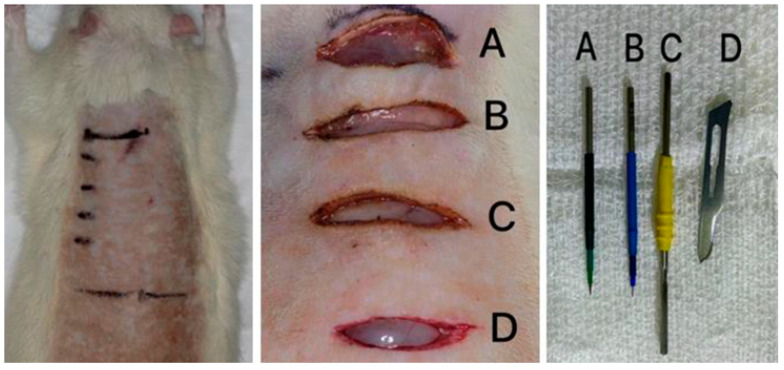
The incisions were made respecting a 10-mm distance between them. At the end, the incisions were sutured by planes with nylon thread. Types of experimental incisions. (A) Needle-type electrode; (B) thin-cut electrode, (C) knife-type electrode; (D) scalpel blade number 15.

Thin-Cut Electrode (PA.02.03.4300), equipped with a microdissection tip (110 × 3 mm) (Traumec, Rio Claro, Brazil). (Figure 2).

Euthanasia was performed with pentobarbital (60 to 100 mg/kg) associated with lidocaine (10 mg/mL), intraperitoneally, after each experimental period. After euthanasia, the area of interest was carefully removed, involving the incisions, to prepare the samples in the experimental periods determined by each group.

### 2.2. Histological Processing

The specimens were first fixed in 10% buffered formalin for 24 h and subsequently submitted to routine histological processing. After embedding in paraffin, 6 μm thick sections of each specimen were divided into slides with six sections each, stained using the hematoxylin and eosin (HE) staining technique or the Picrosirius red staining technique.

### 2.3. Analysis Methods

Histological evaluation was conducted using a DIASTAR light microscope (Leica Reichert & Jung Products, Leica Microsystems GmbH, Wetzlar, Germany) with a magnification range of 5× to 100× and tenfold magnification eyepieces. Images were observed, analyzed, captured, and transferred to a microcomputer for further assessment.

Quantitative analysis was performed on all sections stained with hematoxylin and eosin (HE), taking into account the pattern of inflammatory infiltrate [14,15]. A panoramic image with a 2.5× objective was captured and recorded, covering two peripheral areas and the wound center. Additionally, an image with a 20× objective was obtained for cell counting. Using ImageJ software version 1.53t (National Institutes of Health, Bethesda, MD, USA), the Grid tool in the Plugins tab was applied to create a lattice with 768 intersections. The cell count tool was then used to count only the cells located at the intersection points. The total number of counted points represented the number of cells in the inflammatory infiltrate for each animal.

The samples stained with Picrosirius red were examined under a polarized light optical microscope to determine the concentration of type I and type III collagen fibers [15]. The staining enables a qualitative analysis of collagen fibers and connective tissue by observing variations in color interference, intensity, and birefringence, allowing differentiation between type I and type III fibers. The parameters evaluated included area, density, and the percentage of collagen types I and III [14,15]. Type I collagen fibers appear red, while type III collagen fibers appear green. When the colors overlap, they create a yellowish to orange hue [12]. The photographs were obtained with 2.5× and 20× magnifications, and the analysis was made on 20× magnification. The photomicrographs were analyzed by ImageJ software, measuring the area in μm and in percentage of type I and III collagen. This program lists the percentages by tissue area that were filled by type I and III collagen after staining with Picrosirius red. Initially, the software was calibrated, and, subsequently, images were subjected to individual analysis for type I and type III collagen, using split channel and threshold adjusting images of each group in a single pattern.

### 2.4. Statistical Analysis

As a reference for sample size calculation, a study with similar methodology and outcomes, published by Torres-Augusto Neto et al. (2022) [15], was used. In that study, an estimated error of 5% (*p* < 0.05) and a study power of 20% were established. Based on the detection of a minimum significant difference of 0.5 mm and an average standard deviation of 0.25 mm for histomorphometric evaluation, the estimated sample size was five animals. Considering a projected loss of 20%, the final sample size for each group was set at six animals.

All tabulated data were in continuous quantitative values for cell count, area of different types of collagen (µm), and percentage of the area containing different types of collagen.

The 30 samples were divided into five groups of six individuals according to the cutting material used and for the respective period of time. For each group, Shapiro–Wilk normality and Levene’s homoscedasticity tests were performed. The data obeyed the normal distribution and variances in all groups (*p*  >  0.05). Multiple comparisons were performed for each variable using two-way ANOVA and Tukey’s post-test.

## 3. Results

### Histological and Histomorphometric Analysis

**Figure 3:**
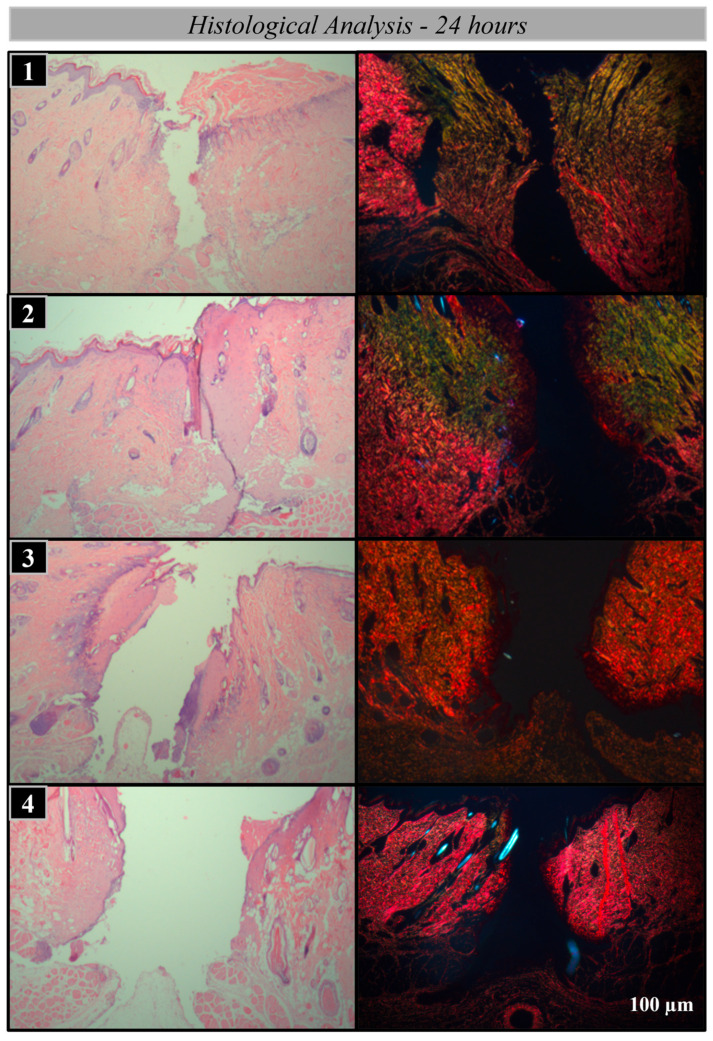
Photomicrograph of HE and Picrosirius red—24 h. 1—Scalpel blade number 15; 2—knife-type electrode; 3—needle-type electrode; 4—thin-cut electrode. Magnification de. 2.5×.

**Figure 4:**
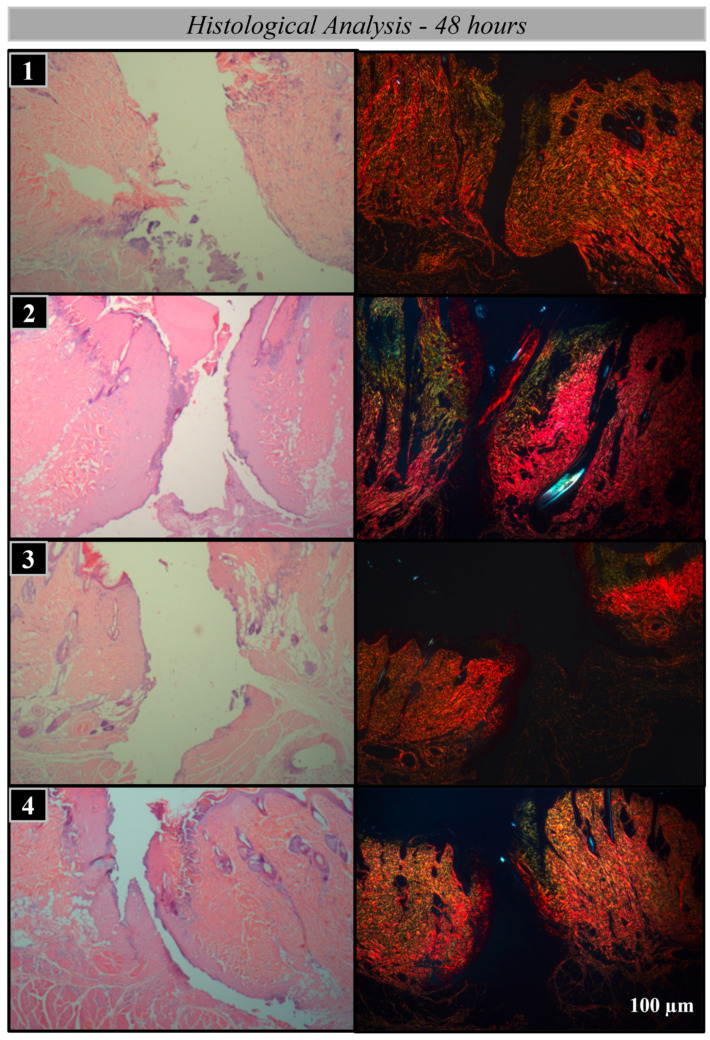
Photomicrograph of HE and Picrosirius red—48 h. 1—Scalpel blade number 15; 2—knife-type electrode; 3—needle-type electrode; 4—thin-cut electrode. Magnification de. 2.5×.

In the histological and histomorphometric analysis at 24 and 48 h, it was observed that the group with the number 15 scalpel blade exhibited an acute inflammatory infiltrate. Regarding collagen, type III fibers were predominant, with a gradual increase in type I collagen. In the knife-type electrode group, considerable tissue damage was observed, characterized by the presence of necrotic and burned tissue. In the needle-type electrode group, notable differences were observed compared to the incisions made with the number 15 scalpel blade; the acute inflammatory process was significantly reduced, and a significant presence of type I collagen was already noticeable at 48 h. Finally, in the thin-cut electrode group, the inflammatory process was similar to that of the conventional scalpel, with maturation of type III collagen occurring within the first 48 h. (Figure 3 and Figure 4).

**Figure 5:**
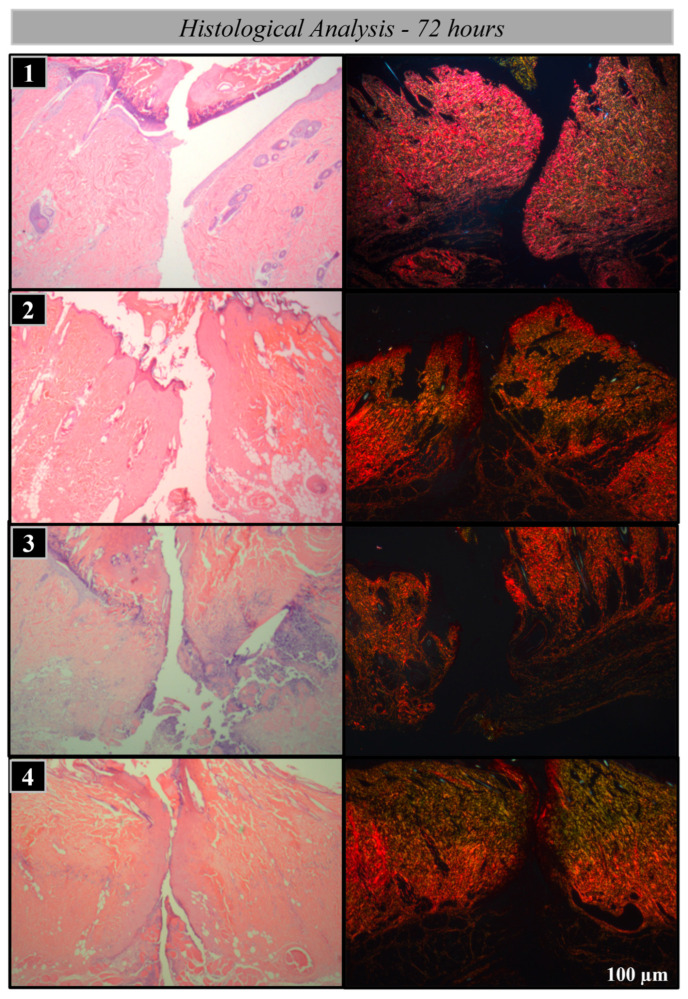
Photomicrograph of HE and Picrosirius red—72 h. 1—Scalpel blade number 15; 2—knife-type electrode; 3—needle-type electrode; 4—thin-cut electrode. Magnification de. 2.5×.

At 72 h, both the number 15 scalpel blade group and the knife-type electrode group exhibited a notably prominent layer of inflammatory cells at the wound margin, with the presence of inflammatory infiltrate in the connective tissue. Type III collagen maturation was initiated. In the needle-type electrode group, the inflammatory process was less pronounced, also showing the beginning of type III collagen maturation. In the thin-cut electrode group, the inflammatory process was minimal, with a significant presence of type I collagen. (Figure 5).

**Figure 6:**
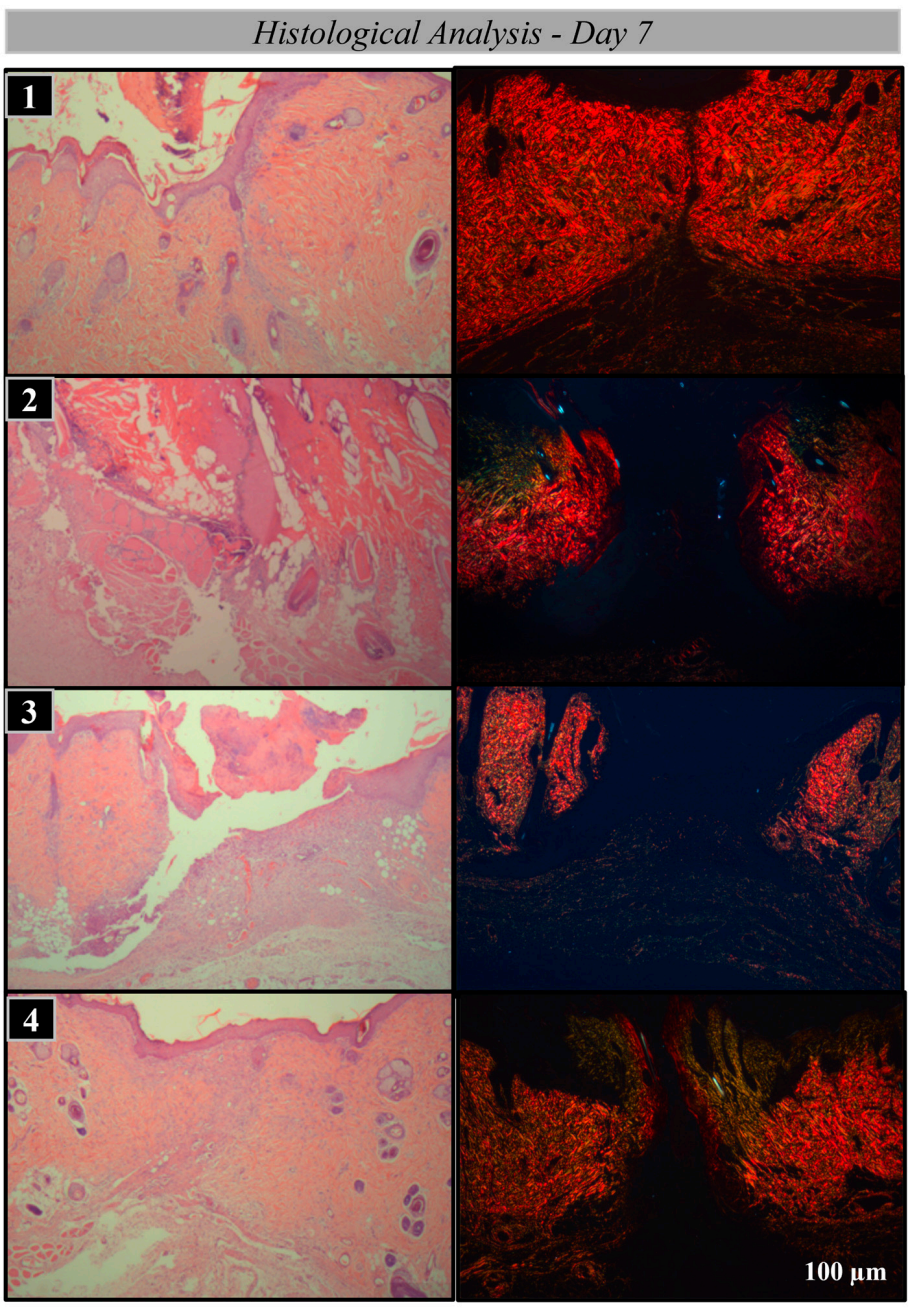
Photomicrograph of HE and Picrosirius red—Day 7. 1—Scalpel blade number 15; 2—knife-type electrode; 3—needle-type electrode; 4—thin-cut electrode. Magnification de. 2.5×.

At 7 days, the number 15 scalpel blade group exhibited increased spacing between connective tissue fibers due to edema. Additionally, the proliferation phase had begun, characterized by crust formation resulting from the coagulation of the exudate. Epithelial cell proliferation was observed in the area of re-epithelialization, leading to wound edge fusion and crust detachment. (Figure 6). At 14 days, during the maturation and remodeling phase, complete regeneration of the superficial dermis and crust detachment were observed. 

**Figure 7:**
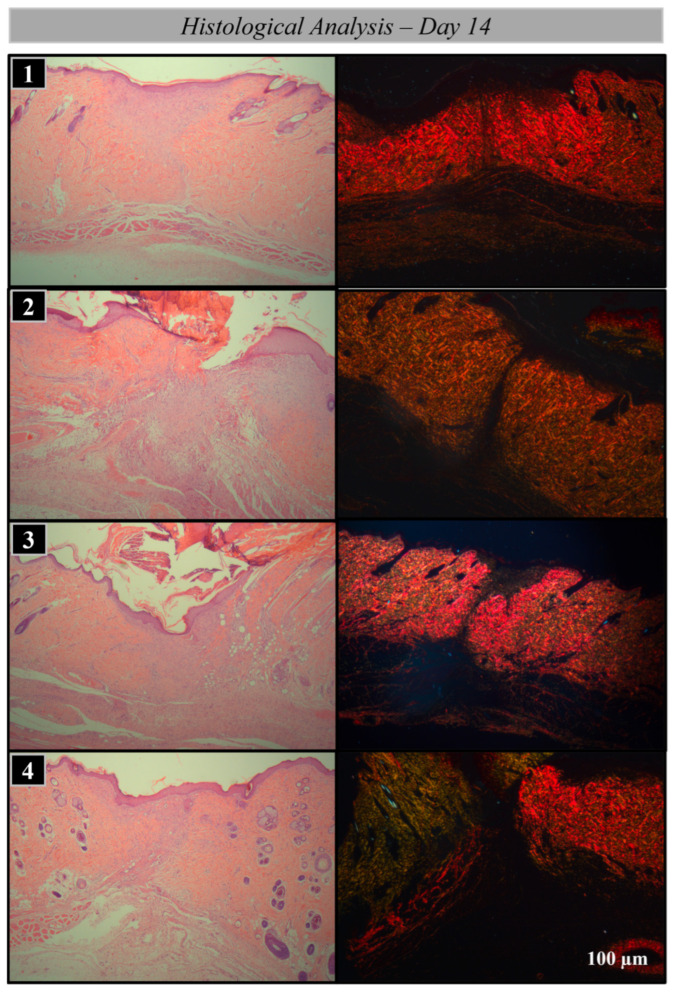
Photomicrograph of HE and Picrosirius red—Day 15. 1—Scalpel blade number 15; 2—knife-type electrode; 3—needle-type electrode; 4—thin-cut electrode. Magnification de. 2.5×.

The knife-type electrode group presented the proliferation phase only at 14 days, indicating a delayed healing process. In the needle-type electrode group, the proliferation phase was observed at an advanced stage, characterized by wound edge fusion, indicating an earlier healing process. The thin-cut electrode group showed complete dermal regeneration and connective tissue maturation. (Figure 7).

**Chart 1:**
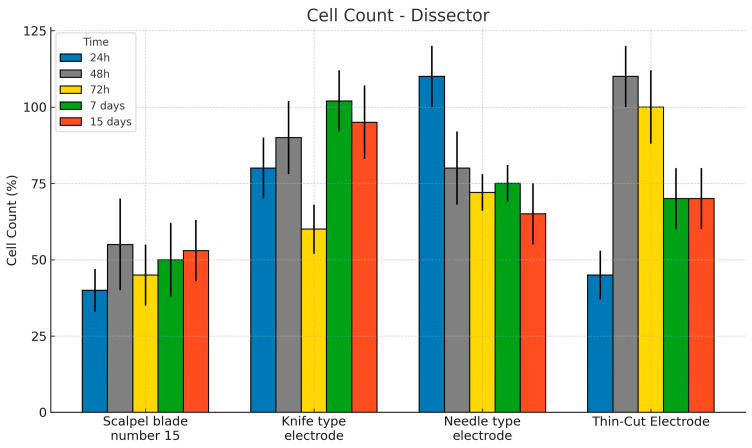
Comparison of cell counts across different dissector types and euthanasia time points. Bars indicate mean values with corresponding standard deviations.

**Chart 2:**
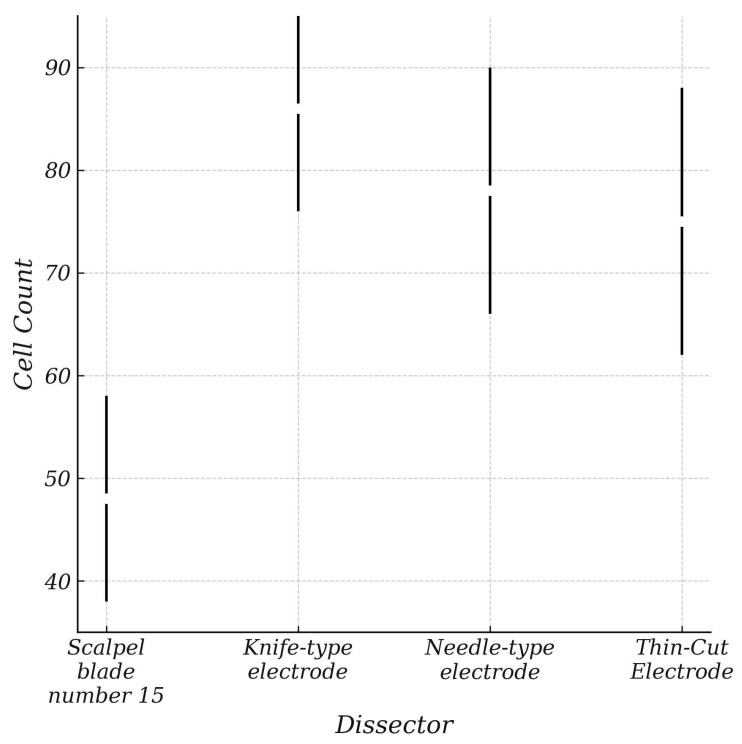
Cell count according to dissector and euthanasia time. The bars represent the mean and standard deviation.

**Chart 3:**
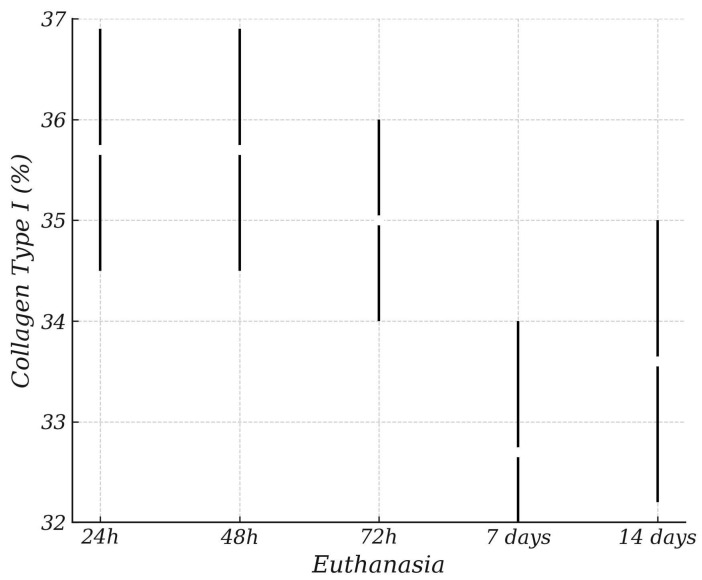
Percentage of type I collagen according to the dissector model. The bars represent the mean and standard deviation.

In the collagen percentage analysis, the highest concentrations of type I collagen were observed in the scalpel blade number 15 and thin cut electrode, microdissection electrode groups, followed by the knife-type electrode and standard microdissection groups. However, the percentage did not show a statistical difference regarding the type of dissector, considered individually, in any of the time points. (Chart 1, Chart 2 and Chart 3).

**Chart 4:**
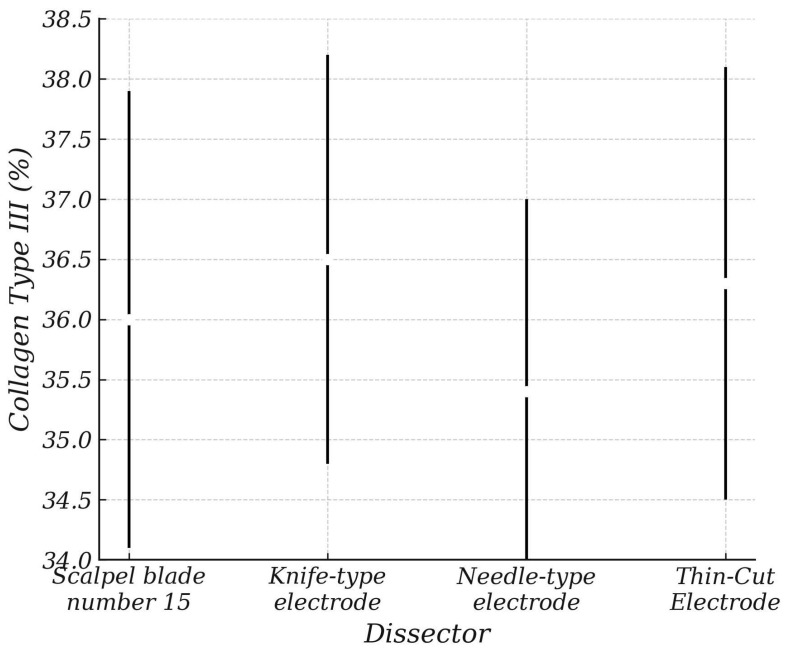
Percentage of type III collagen according to dissector material.

Regarding the percentage of type III collagen, no statistical difference was found between the types of dissectors used, regardless of the euthanasia period analyzed. Overall, in relation to the type of cutting material used, a higher percentage of type III collagen was observed in the incisions made with the knife-type electrode, followed by the thin cut electrode, the scalpel blade number 15, and the needle-type electrode tip, respectively. (Chart 4).

Two-way ANOVA revealed a significant effect of euthanasia time on the percentage of Type I collagen (F(4,99) = 5.198, *p* < 0.001), indicating that collagen maturation varies significantly across the different time points evaluated. Conversely, the type of dissector did not have a significant effect on Type I collagen levels (F(3,99) = 0.603, *p* = 0.615), suggesting that the incision method does not substantially influence collagen type I deposition within the observed timeframe.

Additionally, there was no significant interaction between dissector type and euthanasia time (F(12,99) = 1.048, *p* = 0.412), implying that the pattern of collagen deposition over time is similar regardless of the dissector used.

Post hoc analysis with Tukey’s test confirmed significant differences between specific euthanasia time points, highlighting the progression of collagen maturation during healing. (Table 1).

The estimated marginal means for Type III collagen percentage showed minimal variation among the different dissector types. All groups presented similar mean values, ranging from 35.4% to 36.5%, with overlapping 95% confidence intervals. These findings suggest that the type of dissector used did not significantly influence the deposition of Type III collagen during the evaluated period. (Table 2).

## 4. Discussion

The initial tissue response following injury may vary depending on the dissection technique employed and the type of dissecting instrument used [16,17]. Different dissection modalities influence the release of pro-inflammatory mediators in distinct ways, thereby modulating the early cellular recruitment at the wound site. To assess the progression of healing phases and the rate of tissue repair, a histological evaluation was conducted, focusing on the quantification of phase-specific cellular populations [18]. Picrosirius Red staining was utilized to characterize the composition of the extracellular matrix. In this context, the presence of type III collagen is indicative of the early proliferative phase, marked by disorganized tissue architecture, whereas type I collagen is associated with cellular migration and the later stages of tissue maturation and remodeling [19].

It is well known that scalpel blade injuries are among the most common surgical injuries, second only to suture needle injuries, making the elimination of the scalpel from the operating room increasingly desirable [4,20]. Coagulation electrocautery is frequently used for incising fascia and muscle, and it also contributes to hemostasis, thereby reducing surgical time and blood loss [14].

The results observed in the group treated with the *Thin-cut* electrode were comparable to those of the scalpel blade group, likely due to the reduced contact area with adjacent soft tissues when compared to other thermal devices [14,17]. From a histological perspective, the *thin-cut* group showed lower infiltration of inflammatory cells, which favors a faster repair process and more organized collagen fiber deposition. In contrast, the group treated with the *knife-type* electrode exhibited significantly higher levels of inflammatory cell infiltration, accompanied by delayed collagen renewal and remodeling, suggesting a more intense inflammatory response and a less efficient healing process [15].

Although the scalpel blade provides an excellent tissue response, with minimal trauma, precise incisions, and early healing, its main limitation lies in the lack of hemostasis, which can impair surgical field visibility and prolong operative time [11]. On the other hand, although no statistically significant differences were observed that would indicate inferior clinical outcomes, the microdissection tip demonstrated, through histomorphometric analysis, a performance comparable to that of the scalpel in terms of tissue repair, with the added benefit of causing less tissue damage and promoting intraoperative coagulation [14]. This enhances visualization during the procedure and contributes to reduced surgical time. In comparison, incisions made using the conventional scalpel, traditional electrocautery, and *thin-cut* tip showed distinct responses, with the microdissection tip standing out by combining effective hemostatic control with efficient and well-organized tissue healing—comparable to the best outcomes observed [11,19].

In contrast, the group treated with the *knife-type* electrode exhibited significantly higher levels of inflammatory cells, along with delayed collagen renewal and remodeling, suggesting a more intense inflammatory response and a less efficient healing process [18]. This exacerbated reaction may be attributed to the broader contact surface of the electrode, which leads to greater heat dissipation into adjacent tissues, resulting in more pronounced thermal damage and contributing to slower tissue repair [21].

These findings underscore the importance of carefully selecting the dissection technique and instrument in surgical procedures—not only in terms of cutting efficiency, but also considering the downstream effects on inflammation, tissue organization, and healing dynamics [17]. The microdissection tip, by combining favorable tissue response with effective hemostasis, emerges as a promising alternative to the scalpel blade, particularly in scenarios where improved bleeding control and reduced operative time are critical. Similarly, the *thin-cut* tip demonstrated satisfactory performance, offering effective tissue preservation and reduced initial inflammatory response. In contrast, the outcomes associated with the *knife-type* electrode highlight the need for caution in its clinical application, given the greater thermal injury and its adverse impact on the healing process [20,21,22].

Despite the promising results, this study has some limitations that should be acknowledged. First, the analysis was conducted using an experimental model with controlled biological characteristics, which may not fully replicate the complexity of clinical situations in humans. Additionally, the histological evaluation was performed at specific time points during the healing process, limiting continuous observation of the inflammatory and regenerative dynamics over time. Functional assessments of the regenerated tissues, such as mechanical strength and elasticity, were also not addressed in this study. Future investigations, including long-term clinical and functional evaluations, as well as increasing the sample size and the variety of devices tested, are recommended to validate and expand upon the findings presented here.

## 5. Conclusions

The 15 scalpel blade demonstrated the most favorable wound repair process among the instruments tested. The thin-cut electrode caused, on average, the least tissue damage and showed superior tissue maturation compared with the other electrocautery tips evaluated.

## Figures and Tables

**Table 1:** Two-way ANOVA results for Type I collagen percentage according to dissector type and euthanasia time. Significant differences were further analyzed using Tukey’s post hoc test with adjustments for multiple comparisons.

ANOVA Collagen Type I (%)	Sum of Squares	gl	Mean Square	F	*p*
Dissector	13.8	3	4.59	0.603	0.615
Euthanasia	158.4	4	39.61	5.198	<001
Dissector * Euthanasia	95.8	12	7.99	1.048	0.412
Waste	754.3	99	7.62		

**Table 2:** Estimated marginal means for the percentage of Type III collagen according to dissector type. Values are presented with standard error and 95% confidence intervals.

Estimated Marginal Means Dissector				
			Confidence Interval 95%	
	Mean	Standard Error	Lower Limit	Superior
Knife-type electrode	36.5	0.860	34.8	38.2
Needle-type electrode	35.4	0.860	33.7	37.1
Scalpel blade number 15	36.0	0.877	34.2	37.7
Thin-cut electrode	36.3	0.860	34.6	38.0

## Data Availability

The original contributions presented in this study are included in the article. Further inquiries can be directed to the corresponding author.
